# Evaluating racial and ethnic disparities in antibiotic treatment for pneumonia patients in a major academic health system

**DOI:** 10.1017/ash.2024.472

**Published:** 2024-12-20

**Authors:** David Evans, Kingsley Fortin-Leung, Vishnu Ravi Kumar, Yanru Ma, Radhika Prakash Asrani, Zanthia Wiley, Scott K. Fridkin, CDC Prevention Epicenters Program

**Affiliations:** 1 Department of Epidemiology, Rollins School of Public Health, Emory University, Atlanta, GA, USA; 2 Perelman School of Medicine, University of Pennsylvania, Philadelphia, PA, USA; 3 Division of Infectious Diseases, Department of Medicine, School of Medicine, Emory University, Atlanta, GA, USA; 4 Department of Biostatistics and Bioinformatics, Emory University Rollins School of Public Health, Atlanta, GA, USA

## Abstract

**Objective:**

Examine the relationship between patients’ race and prescriber antibiotic choice while accounting for differences in underlying illness and infection severity.

**Design:**

Retrospective cohort analysis.

**Setting:**

Acute care facilities within an academic healthcare system.

**Patients:**

Adult inpatients from January 2019 through June 2022 discharged from the Hospital Medicine Service with an ICD-10 Code for Pneumonia.

**Methods:**

We describe variability in days of therapy of antimicrobials with activity against *Pseudomonas aeruginosa* (anti-*Pseudomonas* agents) or against MRSA (anti-MRSA agents), by patient’s race and ethnicity. We estimated the likelihood of receipt of any anti-*Pseudomonas* agents by race and modeled the effect of race on rate of use, adjusting for age, severity, and indication.

**Results:**

5,820 patients with 6,700 encounters were included. After adjusting for broad indication, severity, underlying illness, and age, use of anti-*Pseudomonas* agents were less likely among non-Hispanic Black patients than other race groups, although this effect was limited to younger patients (adjusted odds ratio [aOR] 0.45, 95% confidence interval [CI] 0.29, 0.70), and not older ones (aOR 0.98; 95% CI 0.85, 1.13); use of anti-MRSA agents were similar between groups. Among patients receiving any anti-*Pseudomonas* agents, Black patients received them for relatively lower proportion of their inpatient stay (incidence rate ratio 0.91; 95% CI 0.87, 0.96).

**Conclusions:**

We found difference in use of anti-*Pseudomonas* agents between non-Hispanic Black patients and other patients that could not be easily explained by indications or underlying illness, suggesting unmeasured factors may be playing a role in treatment decisions.

## Introduction

Community-acquired pneumonia (CAP) is one of the most common indications for antibiotic prescribing in the inpatient setting.^1–8^ However, antibiotic prescribing for CAP is often inappropriate including excess duration or excessively broad empiric coverage.^1–3^ Antibiotic stewardship (AS) programs aim to reduce guideline-discordant antibiotic prescribing and improve outcomes for patients with CAP.^7–9^ Recently, the American Thoracic Society (ATS) and the Infectious Disease Society of America (IDSA) jointly published guidelines for the management of CAP in 2019, which emphasize use of antimicrobials with activity against *Pseudomonas aeruginosa* or MRSA in a very limited subset of inpatients at highest risk for infection with these pathogens.^10^ Adapting these guidelines to treatment protocols or pathways as part of facility-based antimicrobial stewardship programs are agnostic to a patient’s race.^10^. Several assessments prior to 2019 evaluating race on processes of care for inpatients with pneumonia identified little evidence of race- or ethnicity-based differences in guideline-concordant prescribing for the management of CAP.^11,12^ However, more recent studies suggest race or other social determinants of health (SDH) may affect receipt of antibiotics in different settings.^13–16^ Evaluating SDH factors or race as a driver of disparities in antibiotic prescribing among inpatients is challenging but crucial for ensuring equitable and effective healthcare delivery.^11^ We investigated the effect of race and ethnicity on antimicrobial agent choice and intensity among inpatients with pneumonia in a large Atlanta metropolitan healthcare system.

## Methods

### Study population, data source, and design

We performed a retrospective analysis of inpatients admitted from January 1, 2019, through June 30, 2022, to four acute care hospitals of Emory Healthcare (EHC, Atlanta, GA, USA). These included Hospital A (suburban, non-profit, 582 beds, 46.1% are non-Hispanic Black), Hospital B (urban, non-profit, 537 beds, 71.6% are non-Hispanic Black), Hospital C (suburban, non-profit, 373 beds, 32.4% are non-Hispanic Black), and Hospital D (suburban, non-profit, 152 beds, 19.7% are non-Hispanic Black). Patients eligible for inclusion were adult (≥18 years age) inpatients who received at least one antibiotic during their inpatient hospitalization and were discharged from the hospital medicine service with an International Classification of Diseases, tenth Edition, Clinical Modification (ICD-10-CM) code for pneumonia registered at discharge (i.e., all patients who had one of the following ICD-10 codes either as primary or secondary diagnosis were considered for inclusion: pneumonia J10.0, J11.0, J12.X – J18.x, and J69.X, mycoplasma B96.0, klebsiella B96.1, ornithosis A70.X, and legionellosis A481). Patients admitted for ≥1 day to the intensive care unit were excluded from this analysis to reduce the likely number of providers involved in antibiotic ordering per patient encounter. Roughly 150 hospital medicine faculty worked across the 4 hospitals, including 8 providers working nights exclusively (i.e., nocturnists) and 12 Advanced Practice Providers.

Patient age, sex, race, and ethnicity, ICD-10-CM discharge codes (allowing calculation of Elixhauser score), clinical microbiology data, and cumulative days of antimicrobial agents received for current encounter were extracted from the EHC clinical data warehouse (CDW) and covered all four hospitals. Race values are propagated from historical records within EHR, however per institutional practice, these values can be over-written by patient-provided race values via patient portal inputs at time of encounters. However, which encounters with values provided by patients are unknown. Race and ethnicity were assigned to mutually exclusive groups of Hispanic, non-Hispanic Black, non-Hispanic White, Other, or Unknown based on data entered into the medical record via facility-specific intake procedures.

### Antimicrobial metrics and covariates

Antibiotic use data were generated based on barcode medication administration that had been validated internally and reflect administration during inpatient admission (and exclusive of doses provided in the Emergency Department). Concordance or discordance of choice of agent between Emergency Department and Hospital Medicine Service was not assessed. Each antibiotic administration was mapped to specific dates, and cumulative days of therapy (DOT) for each agent calculated and summed by NHSN defined antibiotic groupings and routes (e.g., IV/PO, exclusion of optic/otic/topical) for each encounter.^17^ We present data for two groups: broad-spectrum hospital-onset infection agents (BS-HO) which we term anti-*Pseudomonas* agents and agents with activity against methicillin-resistant *Staphylococcus aureus* (anti-MRSA agents). The anti-*Pseudomonas* agents in our system consisted mostly of carbapenems, piperacillin/tazobactam, and third- and fourth-generation cephalosporins. For primary analysis, patient-encounters were categorized as either having or having not received any DOT of each antibiotic group. For the secondary analysis, we evaluated intensity of antibiotic exposure among patient encounters in which patients received at least one DOT of anti-*Pseudomonas* agents, expressed as % of patient-days receiving the agents.

Administrative and clinical data were used to create proxy measures of infection severity, comorbid conditions, and established risk factors for pneumonia with *P. aeruginosa*. Severe infections included encounters with ≥1 blood culture positive for any bacteria (bacteremia), sepsis, or co-infection with a urinary tract infection or skin and soft tissue infection (identified through mapped ICD-10 codes on discharge). Comorbidities were summarized by calculating the Elixhauser comorbidity score.^18^ In addition, we generated a second comorbidity score limited to a previously validated subset of conditions that are highly correlated with inpatient antibiotic exposure.^19^ This “antibiotic prone comorbidity score” was left as an ordinal value (0, 1–2, and >2). The later score correlated strongly with the Elixhauser score but performed better in the prediction models and was retained in model building preferentially over the Elixhauser score. Established risk factors for *P. aeruginosa* included any clinical culture growing *P. aeruginosa* in the previous year, any ICD-10 code for cystic fibrosis, and any inpatient hospitalization in the prior 90 days. Established risk factors for MRSA pneumonia were growing MRSA in previous year or inpatient hospitalization in prior 90 days. Note, we had no data on nasal surveillance testing for MRSA to consider in predictive models.

### Statistical approach

Descriptive analysis of characteristics by race was at the encounter level. Using univariate generalized estimating equations (GEE) logistic model to account for patients being hospitalized multiple times during the study’s duration, we estimated the unadjusted risk of receiving anti-*Pseudomonas* agents or anti-MRSA agents (separate models) for each demographic and clinical characteristic. Multivariable GEE logistic regression models, guided by backward selection using the “stepCriterion” function with the “qic” criterion in backward direction to facilitate model selection among variables of interest (having P<.10 on univariate). The analysis of race and ethnicity focused on mutually exclusive groups: Black non-Hispanic, White non-Hispanic, and Hispanic or Latino, with the other races grouped into an “Other” category. Tests for interactions between race and other variables identified significant interactions between age and race. R version 4.3.1 was used for all analyses.

The secondary analysis was limited to patients receiving at least one DOT of anti-*Pseudomonas* agents or anti-MRSA agents (two separate models). Poisson regression modeling was used with length of stay (LOS) as an offset to evaluate the impact of race on intensity of antibiotic use, with calculation of an incidence rate ratio for each racial group. These analyses were limited to encounters between 2 and 14 days of LOS to minimize the impact of overly complicated or prolonged or exceptionally short encounters. Eligible covariates were chosen as described above.

This study was reviewed and approved by the Emory IRB by expedited process under 45 CFR.46.110 and 21 CFR 56.110 because it poses minimal risk and fits expedited review category F[5] as set forth in the Federal Register.

## Results

Eligible patients discharged from Hospital Medicine were from 6,700 encounters with ICD-10 codes for pneumonia and complete demographic data; most patients were non-Hispanic White or non-Hispanic Black (50% and 42%, respectively) with the remaining Hispanic (3%) or other race (5%) (Table 1). When stratified by race and ethnicity, the percentages of the pneumonia encounters with severe infections or co-infections were remarkably similar between groups. Certain comorbid conditions such as renal failure, cardiovascular disease, and the cumulative number of comorbid conditions (sum of antibiotic-prone comorbid conditions) varied slightly by race (Table 1). Factors that potentially influence antibiotic choice were relatively uncommon and varied little by race, included co-infections (urinary tract infections, 16%; skin and soft tissue infections, 2.6%), recent isolation of *P. aeruginosa* from a clinical culture (2.5%), having cystic fibrosis (3.9%), and hospitalization in prior 90 days (13%).

**Table 1.** Characteristics of Inpatient Encounters of Pneumonia Hospitalizations and Discharged by Hospital Medicine Service, by Race & Ethnicity, Emory Healthcare, January 1, 2019, to June 30, 2022

Characteristic, n (%)	Overall (n = 6,700)	Hispanic (n = 183)	Non-Hispanic Black (n = 2,840)	Non-Hispanic White (n = 3,340)	Other (n = 337)
Age (median years) [IQR]	72.0 [58.0, 83.0]	66.0 [48.5, 80.0]	64.0 [49.0, 75.0]	77.0 [67.0, 86.0]	73.0 [62.0, 83.0]
Age >40 years	5,360 (80%)	140 (77%)	2,244 (79%)	2,706 (81%)	270 (81%)
Age ≤40 years	1,340 (20%)	43 (23%)	596 (21%)	634 (19%)	67 (19%)
**Sex**					
Female	3,492 (52%)	95 (52%)	1,583 (56%)	1,674 (50%)	140 (42%)
Male	3,208 (48%)	88 (48%)	1,257 (44%)	1,666 (50%)	197 (58%)
**Infection Severity**					
Bacteremia	781 (12%)	23 (13%)	330 (12%)	394 (12%)	34 (10%)
Sepsis	2,066 (31%)	56 (31%)	852 (30%)	1,042 (31%)	116 (34%)
Urinary tract coinfection	1,064 (16%)	30 (16%)	399 (14%)	588 (18%)	47 (14%)
Skin/soft tissue coinfection	171 (2.6%)	3 (1.6%)	55 (1.9%)	109 (3.3%)	4 (1.2%)
**Underlying illness (UI)**					
Valvular disease	1,363 (20%)	37 (20%)	373 (13%)	873 (26%)	80 (24%)
Peripheral vascular	633 (9.4%)	19 (10%)	242 (8.5%)	347 (10%)	25 (7.4%)
Paralysis	171 (2.6%)	1 (0.5%)	88 (3.1%)	74 (2.2%)	8 (2.4%)
Chronic pulmonary	2,291 (34%)	56 (31%)	948 (33%)	1,202 (36%)	85 (25%)
Diabetes complicated	1,249 (19%)	40 (22%)	684 (24%)	443 (13%)	82 (24%)
Renal failure	1,733 (26%)	36 (20%)	930 (33%)	688 (21%)	79 (23%)
Liver disease	621 (9.3%)	23 (13%)	244 (8.6%)	319 (9.6%)	35 (10%)
**Sum Antibiotic Prone UI**					
None	1,487 (22%)	54 (30%)	539 (19%)	800 (24%)	94 (28%)
1–2	3,937 (59%)	96 (52%)	1,696 (60%)	1,959 (59%)	186 (55%)
>2	1,276 (19%)	33 (18%)	605 (21%)	581 (17%)	57 (17%)
***P. aeruginosa*** **risks**					
*P. aeruginosa* prior year	167 (2.5%)	7 (3.8%)	51 (1.8%)	105 (3.1%)	4 (1.2%)
Cystic fibrosis	262 (3.9%)	14 (7.7%)	99 (3.5%)	141 (4.2%)	8 (2.4%)
Recent hospitalization	854 (13%)	24 (13%)	364 (13%)	433 (13%)	33 (9.8%)
**Insurance Type**					
Private	1,490 (22%)	51 (28%)	721 (25%)	664 (20%)	54 (16%)
Medicaid	936 (14%)	45 (25%)	603 (21%)	206 (6.2%)	82 (24%)
Medicare	4,136 (62%)	80 (44%)	1,450 (51%)	2,415 (72%)	191 (57%)
**Hospital**					
A	2,044 (31%)	59 (32%)	1,014 (36%)	897 (27%)	74 (22%)
B	1,573 (23%)	17 (9.3%)	1,295 (46%)	237 (7.1%)	24 (7.1%)
C	1,358 (20%)	51 (28%)	182 (6.4%)	958 (29%)	167 (50%)
D	1,725 (26%)	56 (31%)	349 (12%)	1,248 (37%)	72 (21%)
**Antibiotic Agents**					
Any anti-Pseudomonas	3,126 (47%)	80 (44%)	1,244 (44%)	1,642 (49%)	160 (47%)
Any anti MRSA	2,779 (41%)	72 (39%)	1,194 (42%)	1,380 (41%)	133 (39%)

In unadjusted GEE modeling, several measures of severity, co-infection, underlying illness, and *P. aeruginosa* risk factors were related to receipt of anti-*Pseudomonas* (Table S1) or anti-MRSA (Table S2) agents. Significant factors with the highest odds ratios associated with receipt in both comparisons included recent hospitalization (OR 1.4 for anti-*Pseudomonas* agents, 1.4 for anti-MRSA), sepsis (OR 2.7 for anti-*Pseudomonas* agents, 3.2 for anti-MRSA), Medicaid or Medicare insurance (OR 1.4 for anti-*Pseudomonas* agents, 1.5 for anti-MRSA) (Tables S1 and S2). Noteworthy was the observation that odds of receipt differed between the hospitals and inconsistently by class of agents (Table S1 and S2).

In an adjusted GEE model, accounting for facility, and insurance status, patients with skin and soft tissue co-infections, diagnosis of sepsis, more antibiotic prone comorbid conditions, and hospitalization in the prior 90 days all were independent predictors of receiving either class of agents (Table 2). Positive *P. aeruginosa* clinical culture in prior year (aOR 7.18; 95% CI 4.28, 12.0) and diagnosis of cystic fibrosis (aOR 1.54; 95% CI 1.12, 2.12) were additional independent predictors of anti-*Pseudomonas* agent use but not anti-MRSA use. Retaining these independent predictors, race was a significant predictor of receipt of anti-*Pseudomonas* agents. Among younger (age ≤40 years) patients with pneumonia, non-Hispanic Black patients (aOR 0.45; 95% CI 0.29, 0.70) and Hispanic patients (aOR 0.38; 95% CI 0.15, 0.93) and had a significantly lower odds of receiving anti-*Pseudomonas* agents compared to other patients (Table 2). This effect was not present among patients over 40 years. Regarding anti-MRSA agents, after adjusting for facility, insurance status, co-infections, severity, comorbidities and recent hospitalization, race was not predictive of receipt of anti-MRSA agents (non-Hispanic Black patients aOR 0.96; 95% CI 0.84, 1.10, and Hispanic patients aOR 0.97; 95% CI 0.70, 1.36) (Table 2).

**Table 2.** Multivariate model estimating independent effect of patient or illness characteristic on receipt of any day of therapy of the anti-pseudomonas agents, or anti-MRSA agents during 6700 inpatient hospitalizations with *Pneumonia* among 5820 patients

	Anti-*P. aeruginosa* agents		Anti-MRSA Agents	
Characteristics	aOR	95% CI	aOR	95% CI
Age <=40				
Non-Hispanic White	Ref			
Hispanic	0.38	0.15, 0.93		
Non-Hispanic Black	0.45	0.29, 0.70		
Other	0.77	0.30, 1.97		
Age >40				
Non-Hispanic White	Ref			
Hispanic	0.91	0.63, 1.30		
Non-Hispanic Black	0.98	0.85, 1.13		
Other	1.13	0.88, 1.46		
All Ages (>18 years)				
Non-Hispanic White				
Hispanic			0.97	0.70, 1.36
Non-Hispanic Black			0.96	0.84, 1.10
Other			0.98	0.76, 1.26
Female	0.88	0.79, 0.99	0.86	0.77, 0.96
Private Insurance^*^	0.71	0.62, 0.81	0.71	0.63, 0.82
Clinical Indications or Co-infections				
Sepsis	2.78	2.48, 3.12	3.29	2.94, 3.68
Urinary tract coinfection	1.68	1.17, 2.41	5.21	3.48, 7.81
Skin/soft tissue coinfection	0.96	0.83, 1.12		
No. of chronic conditions associated with antibiotic use^**^				
None	–	–		
1–2	1.25	1.09, 1.43	1.11	0.97, 1.28
>2	1.61	1.34, 1.93	1.29	1.05, 1.59
Chronic paralysis	2.11	1.47, 3.02	1.46	1.05, 2.04
Diabetes with complications			1.23	1.05, 1.44
Patient history influencing choice				
+ *S. pneumoniae* culture this encounter	0.58	0.27, 1.24		
*+ P. aeruginosa* in past year	7.18	4.28, 12.0	1.29	0.92, 1.82
Cystic cibrosis ICD-10 code	1.54	1.12, 2.12		
Hospitalization in past 90 Days	3.29	2.85, 3.79	2.77	2.41, 3.19

* Adjusting for Facility, baseline patient was government-insurance (Medicaid, Medicare)

** Chronic conditions included: Diabetes, Valvular Disease, Paralysis, Chronic Pulmonary Disease, Peripheral Vascular Disease, Renal Failure, Liver Disease, HIV/AIDS, Lymphoma, Rheumatoid arthritis, obesity, alcohol abuse, and drug abuse.

This difference in antibiotic use by race persisted when evaluating the intensity of antibiotic exposure among the subset of patients receiving some anti-*Pseudomonas* agents. In the Poisson GEE model adjusting for severity, comorbid conditions, and traditional risk factors for *P. aeruginosa* pneumonia, the incidence rate ratio for DOT with anti-*Pseudomonas* agents was 0.91 (95% CI 0.87, 0.96) for non-Hispanic Black patients compared to non-Hispanic White patients (Table S3). This corresponds to 9% fewer DOT with anti-*Pseudomonas* agents during an encounter of similar duration, severity, and comorbid illness. Neither race nor Ethnicity were associated with differences in intensity of anti-MRSA agents after adjusting for relevant factors (Table S3).

## Discussion

We identified a disparity in the choice of antibiotic agents used among inpatients with pneumonia receiving care by Hospital Medicine Service, as well as slight differences in the intensity of treatment defined by the proportion of inpatient days receiving these agents; Black patients were less likely to receive any, and received less intense courses, of agents with activity against *P. aeruginosa*. However, this disparity was mostly limited to younger patients. In contrast, there were no disparity between patients’ race and receipt of agents with activity against MRSA. The inconsistency of finding differences in choice or intensity between patients’ race suggests the etiology of the disparities observed may be nuanced and subtle.

Our data add some supporting evidence that, among younger non-Hispanic Black patients with pneumonia, choice of antibiotic may be influenced by the patient’s race. However, by no means is our study definitive. These findings complement some observations in the pediatric ambulatory setting regarding receipt of any antibiotics for respiratory infections occurring less often among Black patients,^20^ or poorer processes of care among Black inpatients being evaluated for pneumonia.^21^ In our study, the magnitude of the associations with antibiotic choice were very large for key categories of case-mix (e.g., severity, co-infection, risk factors for *P. aeruginosa*) with adjusted odds ratios often >2.0, with the residual association of choice by race limited to patients under 40, a small subset of our patients (roughly 20%). This observation suggests the generalizability of these findings may be muted and require evaluation in a different patient population to better understand its implications. In addition, roughly half of all patients included in this study received agents with anti-*Pseudomonas* activity, such exposure being fairly common with a small difference in crude exposure between race groups. In fact, the crude difference in frequency of receipt between non-Hispanic Black patients and non-Hispanic White patients was small (5 percentage point difference).

A strength of our study was an ability to account for the major drivers of antibiotic class choice including disease severity, co-infection, and traditional risk factors for *P. aeruginosa*; evaluating proxy metrics that directly map to clinical conditions supporting empiric use of agents with anti-*Pseudomonas* activity.^10^ The frequency of these indicators among the study patient encounters was low (roughly 15%) despite frequent use of these agents (roughly 47%). This suggests other drivers of agent choice are likely present and unmeasured. These unmeasured factors may relate to inclusion of some hospital-onset pneumonia, or physician beliefs and attitudes and deserving of ongoing stewardship efforts.^6,13^ The frequency of use of these agents may also be explained by a considerable fraction of patients had ICD-10 codes for more than one infection (i.e., UTI and SSTI), and our evaluation did not account for timing of antibiotic therapy relative to each diagnosis.

At the same time, we should recognize that similar findings were not apparent for exposure to anti-MRSA therapy. The drivers for choosing anti-MRSA therapy mirror in many ways those for anti-*Pseudomonas* therapy among pneumonia inpatients.^10^ However, at these four hospitals, a program to roll out nasal PCR testing for MRSA detection to guide empiric therapy had begun. Perhaps such point-of-care testing reduced or eliminated any race-specific biases in prescriber behavior we may have uncovered regarding use of agents with activity against *P. aeruginosa.* Although the racial disparities in exposure to specific class of antibiotics was subtle, we believe the findings are not by chance. However, the driver of these differences is uncertain; although we could not evaluate the impact of COVID-19 on these findings, the differences observed could be related to differences in managing suspected COVID-19 disease among the younger patient population.

Several limitations are worth noting. First, the data utilized to categorize race, and ethnicity was extracted from documentation in the electronic medical record (EMR) through facility-specific intake procedures. Prior research at other medical centers has shown discordance between races documented in the EMR when compared to patient report.^22,23^ With more complete or accurate data on race and ethnicity, it is possible that our findings would have been different. Also, our infection syndromes were defined based on billing codes (i.e., ICD-10 codes), lacking specificity and sensitivity for definitive clinical infections. Importantly, our cohort likely included roughly 10%–15% of patients inappropriately capture as having pneumonia by ICD-10 codes.^24^ Finally, our inclusion criteria were agnostic to timing of pneumonia, although the exclusion of ICU patients should have minimized the percentage of included patients with hospital-onset pneumonia; we also believe the proportion of disease classified as hospital onset would have been comparable across racial groups.

Overall, our result emphasizes the importance of host factors, severity of illness, and previous clinical cultures with *P. aeruginosa* influencing antibiotic choice. They highlight the significance of individual health conditions and healthcare experiences, while suggesting that patients’ race and ethnicity may have some effect on the classes of antibiotic chosen by the prescriber. These findings are subtle and not consistent across antibiotic classes or patients’ race, ethnicity, or age group. They do demonstrate the necessity for improved ability to acknowledge and mitigate any inherent biases when prescribing antibiotics even in clinical situations where prescribers may believe all decisions are driven solely by clinical severity and indications such as inpatients with pneumonia.

## Supporting information

Evans et al. supplementary material Evans et al. supplementary material

## Supplementary material

For supplementary material accompanying this paper visit http://doi.org/10.1017/ash.2024.472. click here to view supplementary material

## Research transparency and reproducibility

The datasets generated and/or analyzed for the current study are to be available in the Emory Dataverse repository (URL: https://dataverse.unc.edu/dataverse/Emory). To eliminate risk of identification, hospital facility characteristics (bed size, location, etc.) will be suppressed to minimize identification of institution. All personally identifying information (e.g., names, addresses, phone numbers, etc.) are absent from the data in compliance with HHS Guidance Regarding Methods for De-identification of Protected Health Information in Accordance with the HIPAA Privacy Rule.
