# Supplementary material for: Evaluating racial and ethnic disparities in antibiotic treatment for pneumonia patients in a major academic health system
Source: Antimicrob Steward Healthc Epidemiol. 2024 Dec 20;4(1):e221. doi: 10.1017/ash.2024.472 (PMC11696603; doi:10.1017/ash.2024.472)
Supplement: Evans et al. supplementary material [file S2732494X24004728sup001.docx]

| Table S1. Characteristics of pneumonia encounters prescribed with BS-HO antibiotics.  Univariate results from GEE models accounting for correlation within each unique patient for account for multiple hospitalizations | | | | | |
| --- | --- | --- | --- | --- | --- |
|  | Use of Broad Spectrum - Hospital Onset Antibiotics | | Univariate Analysis | | |
| Characteristic | No (n = 3,574) | Yes (n = 3,126) | OR | 95% CI | p-value |
| Age (median years) [IQR] | 72.0 [58.0, 82.0] | 72.0 [59.0, 83.0] | 1.00 | 1.00, 1.00 | 0.5 |
| **Sex** |  |  |  |  |  |
| Female | 1,938 (54%) | 1,554 (50%) | Ref |  |  |
| Male | 1,636 (46%) | 1,572 (50%) | 1.21 | 1.09, 1.34 | <0.001 |
| **Infection Severity** |  |  |  |  |  |
| Bacteremia | 301 (8.4%) | 480 (15%) | 1.89 | 1.63, 2.20 | <0.001 |
| Sepsis | 754 (21%) | 1,312 (42%) | 2.67 | 2.40, 2.97 | <0.001 |
| UTI coinfection | 530 (15%) | 534 (17%) | 1.17 | 1.02, 1.34 | 0.021 |
| SSTI coinfection | 62 (1.7%) | 109 (3.5%) | 2.15 | 1.58, 2.94 | <0.001 |
| **Underlying illness (UI)** |  |  |  |  |  |
| Valvular Disease | 706 (20%) | 657 (21%) | 1.12 | 1.00, 1.26 | 0.060 |
| Peripheral Vascular Disease | 306 (8.6%) | 327 (10%) | 1.26 | 1.07, 1.48 | 0.006 |
| Paralysis | 53 (1.5%) | 118 (3.8%) | 2.45 | 1.78, 3.38 | <0.001 |
| Chronic Pulmonary Disease | 1,204 (34%) | 1,087 (35%) | 0.99 | 0.89, 1.10 | 0.9 |
| Diabetes Complicated | 620 (17%) | 629 (20%) | 1.24 | 1.09, 1.40 | <0.001 |
| Renal Failure | 857 (24%) | 876 (28%) | 1.25 | 1.11, 1.39 | <0.001 |
| Liver Disease | 318 (8.9%) | 303 (9.7%) | 1.10 | 0.93, 1.30 | 0.2 |
| Other Underlying Illness | 1,049 (29%) | 849 (27%) | 0.90 | 0.81, 1.01 | 0.069 |
| Sum Underlying Illness Categories |  |  |  |  |  |
| None | 869 (24%) | 618 (20%) | Ref |  |  |
| 1-2 | 2,083 (58%) | 1,854 (59%) | 1.20 | 1.06, 1.35 | 0.004 |
| >2 | 622 (17%) | 654 (21%) | 1.46 | 1.26, 1.70 | <0.001 |
| ***P. aeruginosa* risk** |  |  |  |  |  |
| *S. pneumoniae* cultured | 18 (0.5%) | 13 (0.4%) | 0.72 | 0.35, 1.46 | 0.4 |
| *P. aeruginosa* prior year | 15 (0.4%) | 152 (4.9%) | 8.51 | 5.67, 12.8 | <0.001 |
| Cystic Fibrosis | 91 (2.5%) | 171 (5.5%) | 1.91 | 1.46, 2.51 | <0.001 |
| Hospitalized Within 90 Days | 350 (9.8%) | 504 (16%) | 1.44 | 1.25, 1.66 | <0.001 |
| **Insurance Type** |  |  |  |  |  |
| Private | 903 (25%) | 587 (19%) | Ref |  |  |
| Medicaid | 492 (14%) | 444 (14%) | 1.36 | 1.15, 1.62 | <0.001 |
| Medicare | 2,110 (59%) | 2,026 (65%) | 1.47 | 1.30, 1.66 | <0.001 |
| Other | 69 (1.9%) | 69 (2.2%) | 1.59 | 1.12, 2.27 | 0.010 |
| **Race and Ethnicity** |  |  |  |  |  |
| Non-Hispanic or Latino White | 1,698 (48%) | 1,642 (53%) | Ref |  |  |
| Hispanic or Latino | 103 (2.9%) | 80 (2.6%) | 0.77 | 0.56, 1.07 | 0.12 |
| Non-Hispanic or Latino Black | 1,596 (45%) | 1,244 (40%) | 0.80 | 0.72, 0.89 | <0.001 |
| Other | 177 (5.0%) | 160 (5.1%) | 0.94 | 0.75, 1.18 | 0.6 |
| **Hospital** |  |  |  |  |  |
| A | 1,008 (28%) | 1,036 (33%) | Ref |  |  |
| B | 978 (27%) | 595 (19%) | 0.61 | 0.53, 0.70 | <0.001 |
| C | 863 (24%) | 495 (16%) | 0.58 | 0.50, 0.67 | <0.001 |
| D | 725 (20%) | 1,000 (32%) | 1.39 | 1.22, 1.59 | <0.001 |

| Table S2. Characteristics of pneumonia encounters prescribed Anti-MRSA antibiotics.  Univariate results from GEE models accounting for correlation within each unique patient to account for multiple hospitalizations. | | | | | |
| --- | --- | --- | --- | --- | --- |
|  | Use of Anti-MRSA Antibiotics | | Univariate Analysis | | |
| Characteristic | No (n = 3,921) | Yes (n = 2,779) | OR | 95% CI | p-value |
| Age (median years) [IQR] | 72.0 [58.0, 83.0] | 71.0 [58.0, 82.0] | 1.00 | 1.0, 1.00 | 0.10 |
| **Sex** |  |  |  |  |  |
| Female | 2,137 (55%) | 1,355 (49%) | Ref |  |  |
| Male | 1,784 (45%) | 1,424 (51%) | 1.26 | 1.14, 1.39 | <0.001 |
| **Infection Severity** |  |  |  |  |  |
| Bacteremia | 172 (4.4%) | 609 (22%) | 5.92 | 4.99, 7.02 | <0.001 |
| Sepsis | 800 (20%) | 1,266 (46%) | 3.23 | 2.91, 3.60 | <0.001 |
| UTI coinfection | 601 (15%) | 463 (17%) | 1.10 | 0.96, 1.26 | 0.2 |
| SSTI coinfection | 34 (0.9%) | 137 (4.9%) | 6.04 | 4.16, 8.77 | <0.001 |
| **Underlying illness (UI)** |  |  |  |  |  |
|  |  |  |  |  |  |
| Valvular Disease | 743 (19%) | 620 (22%) | 1.25 | 1.11, 1.41 | <0.001 |
| Peripheral Vascular Disease | 342 (8.7%) | 291 (10%) | 1.24 | 1.05, 1.46 | 0.012 |
| Paralysis | 77 (2.0%) | 94 (3.4%) | 1.67 | 1.24, 2.26 | <0.001 |
| Chronic Pulmonary Disease | 1,356 (35%) | 935 (34%) | 0.95 | 0.86, 1.05 | 0.3 |
| Diabetes Complicated | 643 (16%) | 606 (22%) | 1.44 | 1.27, 1.63 | <0.001 |
| Renal Failure | 921 (23%) | 812 (29%) | 1.34 | 1.20, 1.51 | <0.001 |
| Liver Disease | 362 (9.2%) | 259 (9.3%) | 1.02 | 0.86, 1.20 | 0.8 |
| Other Underlying Illness | 1,098 (28%) | 800 (29%) | 1.02 | 0.91, 1.14 | 0.7 |
| **Sum Underlying Illness Categories** |  |  |  |  |  |
| None | 942 (24%) | 545 (20%) | Ref |  |  |
| 1-2 | 2,319 (59%) | 1,618 (58%) | 1.18 | 1.04, 1.34 | 0.008 |
| >2 | 660 (17%) | 616 (22%) | 1.60 | 1.37, 1.87 | <0.001 |
| ***P. aeruginosa* risk** |  |  |  |  |  |
| *S. pneumoniae* cultured | 11 (0.3%) | 20 (0.7%) | 2.34 | 1.13, 4.86 | 0.023 |
| *P. aeruginosa* prior year | 73 (1.9%) | 94 (3.4%) | 1.74 | 1.28, 2.37 | <0.001 |
| Cystic Fibrosis | 147 (3.7%) | 115 (4.1%) | 1.12 | 0.85, 1.46 | 0.4 |
| Hospitalized Within 90 Days | 423 (11%) | 431 (16%) | 1.38 | 1.19, 1.60 | <0.001 |
| **Insurance Type** |  |  |  |  |  |
| Private | 978 (25%) | 512 (18%) | Ref |  |  |
| Medicaid | 517 (13%) | 419 (15%) | 1.53 | 1.29, 1.82 | <0.001 |
| Medicare | 2,353 (60%) | 1,783 (64%) | 1.45 | 1.28, 1.64 | <0.001 |
| Other | 73 (1.9%) | 65 (2.3%) | 1.74 | 1.22, 2.48 | 0.002 |
| **Race and Ethnicity** |  |  |  |  |  |
| Non-Hispanic or Latino White | 1,960 (50%) | 1,380 (50%) | Ref |  |  |
| Hispanic or Latino | 111 (2.8%) | 72 (2.6%) | 0.89 | 0.65, 1.23 | 0.5 |
| Non-Hispanic or Latino Black | 1,646 (42%) | 1,194 (43%) | 1.02 | 0.92, 1.13 | 0.8 |
| Other | 204 (5.2%) | 133 (4.8%) | 0.93 | 0.74, 1.18 | 0.6 |
| **Hospital** |  |  |  |  |  |
| A | 1,104 (28%) | 940 (34%) | Ref |  |  |
| B | 953 (24%) | 620 (22%) | 0.76 | 0.67, 0.88 | <0.001 |
| C | 911 (23%) | 447 (16%) | 0.58 | 0.50, 0.67 | <0.001 |
| D | 953 (24%) | 772 (28%) | 0.96 | 0.84, 1.10 | 0.6 |

Table S3. Multivariate Poisson Model Estimating Independent Effect of Race on proportion of the patient’s dates of inpatient stay receiving antibiotics, by antibiotic grouping, among patients with pneumonia receiving at least one day of therapy for the specified group. Analysis was limited to 2,320 patients with 2,653 hospitalizations between 2 and 14 days.

|  | Anti-*P. aeruginosa* agents | |  | Anti-MRSA Agents | |
| --- | --- | --- | --- | --- | --- |
| Characteristics | IRR | 95% CI |  | IRR | 95% CI |
| **Race and Ethnicity** |  |  |  |  | |
| Non-Hispanic White | — | — |  | — | — |
| Hispanic | 1.07 | 0.95, 1.20 |  | 1.13 | 0.89, 1.44 |
| Non-Hispanic Black | 0.91 | 0.87, 0.96 |  | 0.96 | 0.88, 1.05 |
| Other | 1.04 | 0.94, 1.15 |  | 0.89 | 0.74, 1.07 |
| Female | — | — |  | 0.84 | 0.78, 0.90 |
| Clinical Indications or Co-infections |  |  |  |  |  |
| Sepsis | 1.05 | 1.00, 1.09 |  | 1.29 | 1.20, 1.39 |
| SSTI | — | — |  | 1.51 | 1.30, 1.75 |
| UTI | — | — |  | 0.81 | 0.73, 0.91 |
| No. of chronic conditions associated with antibiotic use** |  |  |  |  |  |
| None | - |  |  | — | — |
| 1-2 | 0.87 | 0.79, 0.96 |  | 0.88 | 0.80, 0.96 |
| >2 | 0.93 | 0.87, 0.99 |  | 0.94 | 0.82, 1.08 |
| Liver Failure | — | — |  | 0.67 | 0.59, 0.78 |
| Chronic obstructive pulmonary disease | 1.10 | 1.04, 1.16 |  | — | — |
| Chronic Renal Failure | 0.92 | 0.87, 0.98 |  | 0.80 | 0.72, 0.88 |
| Patient history influencing choice |  |  |  |  |  |
| *S. pneumoniae* cultured | 0.87 | 0.64, 1.18 |  | — | — |
| *P. aeruginosa* in past year | 1.22 | 1.12, 1.33 |  | — | — |
| Cystic Fibrosis | 1.35 | 1.22, 1.49 |  | — | — |
| Hospitalization in past 90 Days | 1.06 | 1.02, 1.11 |  | 1.18 | 1.09, 1.28 |

*Incidence rate ratio (IRR) Adjusting for Facility

**Chronic conditions included : Diabetes, Valvular Disease, Paralysis, Chronic Pulmonary Disease, Peripheral Vascular Disease, Renal Failure, Liver Disease, HIV/AIDS, Lymphoma, Rheumatoid arthritis, obesity, alcohol abuse, drug abuse

Table S4. CDC NHSN Antibiotic Use Option Groups

Adult broad spectrum antibacterial agents predominantly used for hospital-onset infections

(BS-HO):

- Amikacin (IV only)
- Aztreonam (IV only)
- Cefepime
- Ceftazidime
- Doripenem
- Gentamicin (IV only)
- Imipenem/Cilastatin
- Meropenem
- Piperacillin/Tazobactam
- Tobramycin (IV only)

Adult Antibacterial agents predominantly used for resistant Gram-positive infections (e.g.,MRSA):

- Ceftaroline
- Dalbavancin
- Daptomycin
- Linezolid
- Oritavancin
- Quinupristin/Dalfopristin
- Tedizolid
- Telavancin
- Vancomycin (IV only)

Adult broad spectrum antibacterial agents predominantly used for community-onset infections (BS-CO):

- Amoxicillin/Clavulanic Acid (Augmentin)
- Cephalexin (Keflex)
- Trimethoprim/Sulfamethoxazole (Bactrim, Septra)
- Doxycycline
- Ciprofloxacin (Cipro)
- Levofloxacin (Levaquin)
- Azithromycin (Zithromax)
- Clindamycin
- Penicillin (Pen VK)
- Ceftriaxone (Rocephin)
